# An In Silico Analysis Reveals Sustained Upregulation of Neuroprotective Genes in the Post-Stroke Human Brain

**DOI:** 10.3390/brainsci13070986

**Published:** 2023-06-23

**Authors:** Federica Betto, Luigi Chiricosta, Emanuela Mazzon

**Affiliations:** IRCCS Centro Neurolesi “Bonino-Pulejo”, Via Provinciale Palermo, Contrada Casazza, 98124 Messina, Italy

**Keywords:** ischemic stroke, post-stroke, in silico analysis, transcriptome, neuroprotection, neuroinflammation, oxidative stress, human brain

## Abstract

Ischemic stroke is a cerebrovascular disease caused by an interruption of blood flow to the brain, thus determining a lack of oxygen and nutrient supply. The ischemic event leads to the activation of several molecular signaling pathways involved in inflammation and the production of reactive oxygen species, causing irreversible neuronal damage. Several studies have focused on the acute phase of ischemic stroke. It is not clear if this traumatic event can influence some of the molecular processes in the affected area even years after the clinical event. In our study, we performed an in silico analysis using freely available raw data with the purpose of evaluating the transcriptomic state of post-mortem brain tissue. The samples were taken from non-fatal ischemic stroke patients, meaning that they suffered an ischemic stroke and lived for a period of about 2 years after the event. These samples were compared with healthy controls. The aim was to evaluate possible recovery processes useful to mitigating neuronal damage and the detrimental consequences of stroke. Our results highlighted differentially expressed genes codifying for proteins along with long non-coding genes with anti-inflammatory and anti-oxidant functions. This suggests that even after an amount of time from the ischemic insult, different neuroprotective mechanisms are activated to ameliorate brain conditions and repair post-stroke neuronal injury.

## 1. Introduction

Worldwide, stroke is one of the most dangerous cerebrovascular events, even causing permanent disabilities and death [1,2].

Stroke is a multifactorial disease characterized by several risk factors such as age, sex, ethnicity, and genetics [3]. Other major risks are hypertension, hypercholesterolemia, carotid stenosis, and atrial fibrillation. In addition, cigarette smoking, alcohol, diabetes and insulin resistance are factors predisposing to this condition [4].

Stroke is defined as ischemic when an interruption of blood flow to the brain tissue occurs. Ischemic stroke determines deprivation of nutrients and oxygen supply to the affected area. The event triggers a cascade of molecular responses that can cause metabolic and molecular alteration, leading to irreversible neuronal impairment [2].

A common secondary mechanism as a consequence of ischemic stroke is neuroinflammation [5].

The principle anti-inflammatory mechanism is exerted by microglial cells. Indeed, after ischemic insult, microglia release cytokines, recruit immune cells and break protein aggregates along with dead cells [6]. Nevertheless, at the same time, the release of pro-inflammatory cytokines could determine cerebral tissue damage and neurodegeneration [7,8].

Following the interruption of blood flow, hypoxia leads to an increase in H^+^ concentration and mitochondrial dysfunction, promoting the production of reactive oxygen species (ROS) and worsening the oxidative process. In the post-stroke brain, astrocytes and glial cells are activated to protect the central nervous system from oxidative damage through antioxidant processes, such as Nrf2 activation (nuclear factor erythroid 2-related factor 2) [9]. The event promotes the transcription of anti-oxidant factors such as heme oxygenase 1 (HO-1), NADPH-quinone oxidoreductase (NOQ1), and glutathione biosynthesis [10]. Previous evidence demonstrates that oxidative stress contributes to neuronal damage after ischemia–reperfusion injury [11] and that inhibition of this process protects against neurodegeneration [12].

Neuroprotection after ischemic stroke has been studied in the last few years and some factors have been highlighted as neuroprotective: besides Nrf2 [13], other factors such as brain-derived neurotrophic factor (BDNF), which is important for neurogenesis and neuronal plasticity [14], and hypoxia-inducible factor-1α (HIF-1α), which mediates neurogenesis and angiogenesis and activates autophagy by microglial cells [15].

One of the therapeutic strategies used to treat ischemic stroke is reperfusion which consists of re-opening the obstructed vessels to promote the restoration of blood flow and re-oxygenation of the brain. However, in some cases, reperfusion can paradoxically exacerbate tissue injury and inflammation, worsening neuronal suffering and damage. This event, defined as “ischemia–reperfusion injury”, could trigger different biological responses such as both adaptative and immune responses or cell death [16,17]. Reperfusion injury contributes to several pathological conditions, such as oxidative stress, inflammation, metabolic disorders, and cytokines damage [18].

Unfortunately, most of the studies that have focused on ischemic stroke, until now, took advantage of either in vitro or in vivo models. Animal experimental models employed by the scientific community are useful to understand the basal mechanisms that underlie cerebral ischemia and recovery. For instance, transcriptomic inspection was one of the strategies used to investigate stroke conditions in the model. Several long non-coding RNAs were observed to be deregulated, and their upregulation was linked to cell death, autophagy, inflammation, angiogenesis, and infarction [19]. Additionally, recent studies have implemented single-cell strategies to study the role of specific cell populations involved in stroke through the study of the transcriptomic profile. The studies mainly highlighted the role of microglia, astrocytes, and oligodendrocytes in inflammation and stress along with neurons in cell differentiation and neurogenesis [20].

Nevertheless, not all aspects of human stroke can be explored in animals because of the complex pathophysiology of the disease [21]. Moreover, animal models are useful to study acute ischemic insult but not to evaluate the consequences of the stroke and recovery mechanisms of the brain years after the event [22].

To our knowledge, due to the difficulty of studying stroke in human samples, very few experiments are in the literature.

Specifically, we focused our attention on RNA-seq experiments that inspect the brain tissue of human samples with a stroke event that occurred during their life. The Gene Expression Omnibus (GEO) repository [23] of the National Center for Biotechnology Information (NCBI) shows that only the work of Huttner H.B. et al. is associated with human brain stroke for RNA-seq for this purpose. The authors of the work focused their attention on the transcriptome of the cortical tissues of stroke patients in comparison to non-stroke individuals to search for gene fusion events [24]. Nevertheless, they did not observe any genomic alteration able to clarify the stroke condition.

Taking advantage of the same cohort made of stroke and healthy individuals, the aim of our analysis was, instead, the change in the level of expression of the transcriptomic profiles due to non-fatal stroke conditions in order to focus on the recovery of the brain tissue. Specifically, the non-fatal ischemic event itself is not the declared cause of death of these patients who lived for an average of 2.6 years after the event.

Thus, our in silico analysis was performed using raw data from the transcriptomic analysis of post-mortem tissue samples of patients who suffered from a non-fatal ischemic stroke during their life.

## 2. Materials and Methods

### 2.1. Data Collection

Raw data were obtained from the GEO repository with the Sequence Read Archive (SRA) project code PRJNA242801. The dataset is composed of 13 runs among which 6 are associated with a healthy condition and 7 are associated with a cortical ischemic stroke condition. The original paper of Huttner H.B. et al. specifies that the patients with a stroke died because of other conditions a while after the stroke occurred. All tissues were retrieved from the Departments of Neuropathology at the Universities of Debrecen and Lund in accordance with the local ethics committee.

### 2.2. Cohort Description

After the principal component analysis (PCA), we reduced the cohort to 10 patients, 5 stroke patients and 5 non-stroke subjects matched for age and gender, as declared by the authors. All of them suffered from a stroke located in the middle cerebral artery and samples were retrieved surrounding that area. The mean age of the cohort is 76.40 with a standard deviation of 6.35. As reported in Table 1, the mean age of death after stroke was 2.6 years with a standard deviation of 1.52. The causes of death were different from patient to patient.

### 2.3. Bioinformatics Inspection

The raw data were checked for quality using the fastqc tool version 0.11.9 from the Babraham Institute in Cambridge, UK. Trimmomatic 0.40-rc1 version (Usadel Lab, Aachen, Germany) was used to eliminate adapters and low-quality bases [25]. The cleaned reads were then aligned to the human reference genome (GRCh38) using the STAR RNA-seq aligner 2.7.10a_alpha_220207 (New York, NY, USA) [26]. The htseq-count python package version 0.13.5 (European Molecular Biology Laboratory (EMBL), Heidelberg, Germany) was used to compute the expression levels of the transcripts [27]. Low-expressed transcripts (<10 counts) were removed a priori to increase the power of the analysis. Differentially expressed genes (DEGs) were identified using the DESeq2 library (version 1.36.0) [28] in R version 4.2.0 (R Core Team), and false positive DEGs were removed using the Benjamini–Hochberg procedure with a q-value of 0.05. The enrichment of the Biological Process terms of the Gene Ontology was performed in R using the biomaRt package version 2.52.0.

## 3. Results

### 3.1. Cohort Selection

**Figure 1:**
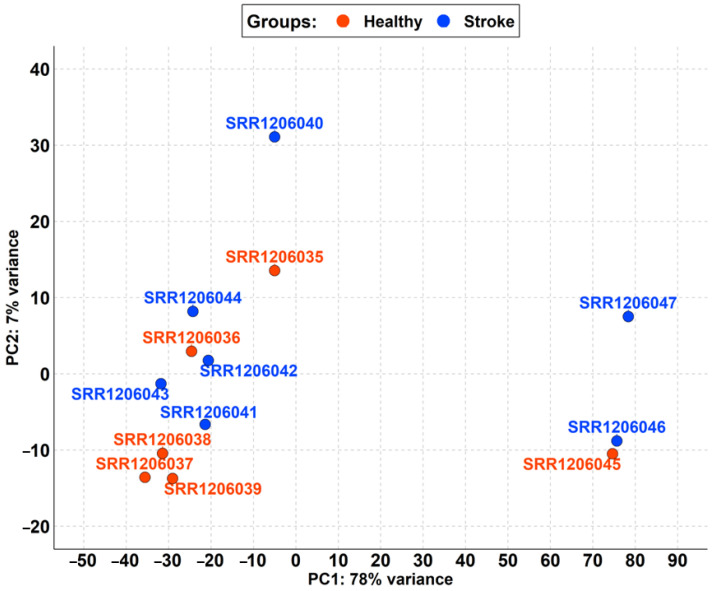
PCA of samples retrieved in PRJNA242801 differentiated by the healthy (red) and stroke (blue) groups. PC1 strongly splits the SRR1206045, SRR1206046, and SRR1206047 samples from the remaining samples, highlighting a variance of 78%. Instead, the PC2 cluster split all of the samples quite well even if SRR1206040 is a bit of a distance away but with a small variance of just 7%.

The focus of the first step of the analysis was the inspection and evaluation of the distribution of the 13 samples. In detail, we checked the homogeneity of the raw data in the groups of healthy and stroke. Thus, we realized the PCA that is plotted in Figure 1.

Among the healthy and stroke samples, the first component of PCA (PC1) highlights a variance of 78% due to the high distance of the SRR1206045, SRR1206046, and SRR1206047 samples (Figure 1). The second component (PC2) has only 7% variance.

**Figure 2:**
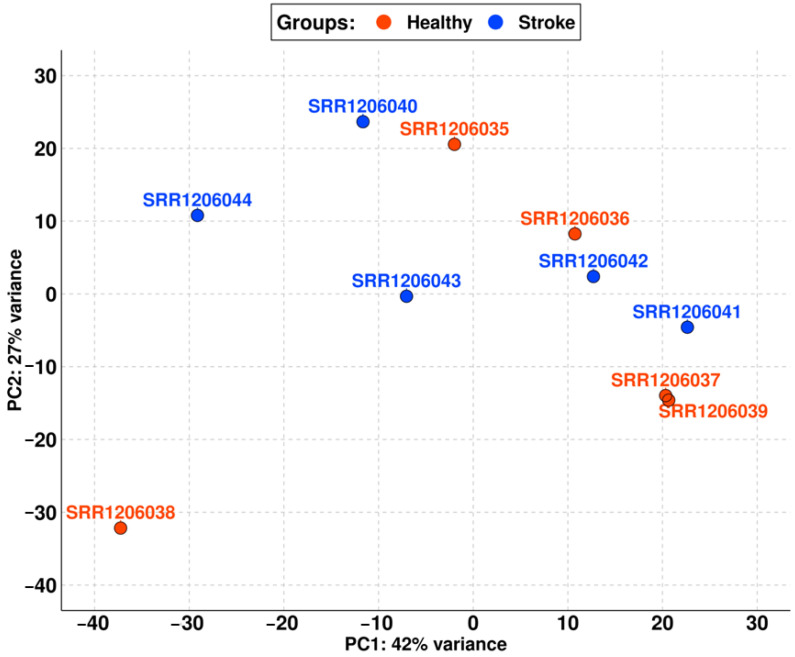
PCA of the final cohort with samples differentiated into the healthy (red) and the stroke (blue) groups. PC1 has very little variance between the samples (only 42%), so the two groups are comparable.

For this reason, we removed the aforementioned samples from the analysis to clear the dataset. In Figure 2, we reported the new PCA obtained with the remaining runs, which shows that the difference between the stroke and non-stroke samples is very low. Indeed, PC1 has only 42% variance, so the two groups are comparable.

Thus, the final cohort of our study is composed of five elements for the healthy group (SRR1206035, SRR1206036, SRR1206037, SRR1206038, and SRR1206039) and five elements for the stroke group (SRR1206040, SRR1206041, SRR1206042, SRR1206043, and SRR1206044).

### 3.2. Comparative Transcriptomic Analysis

**Figure 3:**
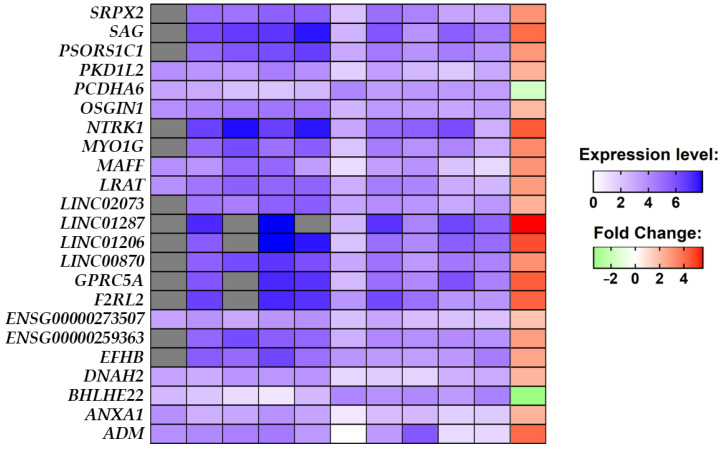
The expression level of each of the 23 DEGs identified in the comparative analysis between the healthy and stroke groups for each sample in the blue palette. The expression level of the counts was scaled from 0 to 1 and then normalized in a log scale of the normalized expression level count. Transcripts not expressed in a sample are painted in gray. From left to right the blue palettes represent samples SRR1206035, SRR1206036, SRR1206037, SRR1206038, SRR1206039, SRR1206040, SRR1206041, SRR1206042, SRR1206043, and SRR1206044. The comparative analysis shows upregulated DEGs in the red palette and downregulated DEGs in the green palette.

In the final cohort, composed of five stroke patients and five non-stroke individuals, we performed a comparative analysis between the stroke group against the non-stroke group which highlighted the 23 DEGs. The heatmap in Figure 3, along with the expression level identified for each sample, shows that 21 out of the 23 DEGs are upregulated, while *BHLHE22* and *PCDHA6* are downregulated in stroke conditions. What is noteworthy is that all of the DEGs, except for *ENSG00000273507* and *PCDHA6*, have a very strong alteration in fold change by at least two orders of magnitude.

Among all DEGs, ADM, *ANXA1*, *BHLHE22*, *DNAH2*, *EFHB*, *F2RL2*, *GPRC5A*, *LRAT*, *MAFF*, *MYO1G*, *NTRK1*, *OSGIN1*, *PCDHA6*, *PKD1L2*, *PSORS1C1*, *SAG*, and *SRPX2* are protein-coding genes, *ENSG00000259363* and *ENSG00000273507* contain an open reading frame, and *LINC00870*, *LINC01206*, *LINC01287* and *LINC02073* are long intergenic non-coding transcripts. The mean normalized expression level for each group is reported in Table 2 along with its significance level.

The enrichment analysis computed against the Gene Ontology dictionary identified eight identified ontologies among which “G protein-coupled receptor internalization” (GO:0002031), “desensitization of G protein-coupled receptor signaling pathway“ (GO:0002029), “negative adaptation of signaling pathway“ (GO:0022401), “adaptation of signaling pathway“ (GO:0023058), “positive regulation of cell migration involved in sprouting angiogenesis” (GO:0090050), “detection of external stimulus“ (GO:0009581), and “detection of abiotic stimulus“ (GO:0009582) are biological process terms while “phagocytic cup“ (GO:0001891) is a cellular component term. In detail, *ADM* and *SAG* enrich GO:0002031, GO:0002029, GO:0022401 and GO:0023058, *ANXA1* and *SRPX2* enrich GO:0090050, and *NTRK1*, *PKD1L2* and *SAG* enrich GO:0009581 and GO:0009582, while *ANXA1* and *MYO1G* enrich GO:0001891. Interestingly, no one KEGG pathway was enriched.

## 4. Discussion

Ischemic stroke is a pathological condition caused by the transitory interruption of blood supply to the brain leading to irreversible neuronal damage. Nowadays, the scientific literature shows interest in understanding the cause of brain injuries along with the length of recovery after the traumatic event [29]. In this line, the aim of this work was to understand the possible role of the proteins and factors involved in processes that can be considered beneficial for post-ischemic brain recovery and that endure for a long-time after the event.

For this purpose, we chose from the online database SRA the project code PRJNA242801. This project collects the transcriptomic profile of brain tissue of patients with an ischemic stroke event during their lives or non-stroke individuals obtained by the RNA-seq strategy. To our knowledge, no other raw data of brain samples have been deposited in this databank both from stroke and non-stroke individuals.

As shown in Table 1, the cohort of stroke samples has a mean age of 76.40. No patient died because of an acute stroke but all of them survived at least one year after the ischemic event. Also, all of them died from other causes that differ from patient to patient. The non-stroke cohort matches the stroke cohort both for gender and age. Nevertheless, the PCA in Figure 1 shows that not all the samples could be used in the analysis. Indeed, the SRR1206045, SRR1206046, and SRR1206047 samples were quite distant from the others probably due to biological variance. After removing them, the PCA in Figure 2 highlights the high level of comparability among the two cohorts and among the samples of each cohort itself.

What is noteworthy is that Huttner H.B. et al. [24], the authors of the original manuscript, used RNA-seq to focus their attention on the analysis of different fusion genes. Herein, we took advantage of the transcriptomic data to perform a comparative analysis between stroke and non-stroke individuals.

The final result of our comparative analysis was very interesting, whereby it highlighted that, in the whole transcriptome, only 23 DEG results differed significantly between the non-stroke and stroke brain samples (Figure 3). Specifically, the Table 2 shows that *ADM*, *ANXA1*, *DNAH2*, *EFHB*, *ENSG00000259363*, *ENSG00000273507*, *F2RL2*, *GPRC5A*, *LINC00870*, *LINC01206*, *LINC01287*, *LINC02073*, *LRAT*, *MAFF*, *MYO1G*, *NTRK1*, *OSGIN1*, *PKD1L2*, *PSORS1C1*, *SAG*, and *SRPX2* are upregulated and only *BHLHE22* and *PCDHA6* are downregulated.

The examined data showed strong up-regulation of *ADM*, encoding for adrenomedullin. Adrenomedullin is a neuroprotective peptide, secreted widely in the central nervous system after cerebral ischemia as a response to hypoxia [30,31]. Adrenomedullin has a vasodilatory effect; it is able to regulate blood–brain barrier (BBB) functionality [32] and protect the brain against ischemia–reperfusion injury by stimulating glial cell survival and migration [33]. Moreover, adrenomedullin is able to promote angiogenesis in the ischemic brain increasing nitric oxide (NO) synthesis and stimulating vascular regeneration in the infarct area [33,34]. The high expression of *ADM* in the samples analyzed led us to hypothesize that adrenomedullin was secreted in the brain of the affected subjects and is still secreted years after the ischemic stroke. The upregulation of *ADM* in the results suggests its neuroprotective role and its capacity to promote vascular recovery even years after the ischemic event.

Another gene that resulted in being upregulated is the *NTRK1* gene that codifies for the tropomyosin receptor kinase A (TrkA) receptor. This latter gene is the high-affinity receptor for the nerve growth factor (NGF), involved in neural development. TrkA, interacting with its ligand NGF, dimerizes and auto-phosphorylates its tyrosine residues, activating the downstream pathway that can lead to proliferation and differentiation [35]. Evidence shows that TrkA engagement promotes the viability and migration of cells in an experimental model of ischemic stroke [36]. Furthermore, another ligand of the TrkA receptor is neurotrophin-3, which has been studied to attenuate immune inflammatory response after stroke and limit cell death [37]. Previous evidence has shown TrkA activation is important in promoting neuronal survival and growth, in particular in the context of oxidative stress processes. In fact, downstream signaling of TrkA attenuates oxidative damage [38]. The antioxidant effect of TrkA is also exerted through direct involvement in the synthesis pathway of glutathione (GSH), an essential cellular antioxidant molecule [39]. Considering that the production of ROS is one of the processes present in ischemic stroke, the strong upregulation of *NTRK1* in our data suggests that TrkA could have a neuroprotective role against oxidative-species-induced damage even after an average of 2.5 years from the ischemic event.

Herein, we observed the upregulation of the *MAFF* and *OSGIN1* genes, both codifying for proteins related to the Nrf2 transcription factor. The small musculoaponeurotic fibrosarcoma oncogene homolog F (MafF), codified by the *MAFF* gene, belongs to a family of basic leucine zipper transcription factors implicated in different neurological disorders [40,41]. *MAFF* is induced by hypoxia, and it is strongly responsive to oxidative stress. Its role is exerted by forming a heterodimer with the Nrf2 transcription factor, which is one of the most important regulators of antioxidant response, and then binding to the antioxidant response elements (AREs) on the DNA [42].

*OSGIN1* (also called *OKL38*) codifies oxidative-stress-induced growth inhibitor 1 (OSGIN1). Despite little being known about OSGIN1’s mechanism of action, it has been demonstrated that OSGIN1 is regulated by Nrf2 in response to oxidative stress stimuli in human astrocytes [43], and it has been proven that OSGIN1 mediates astrocytes’ protection against hydrogen peroxide (H_2_O_2_)-induced injury [44].

The proteins codified by the genes described above are involved in the response to oxidative stress which is one of factors the responsible for the detrimental consequences of ischemic stroke. In fact, reperfusion treatment often leads to free radical production, protein oxidation, and DNA damage [11]. In addition, oxidative processes induce the release of damage-associated molecular patterns (DAMPs), triggering the inflammatory response and worsening the severity of damage [45]. Furthermore, oxidative stress’ negative effects are also associated with vascular dysfunction and blood–brain barrier alteration, promoting disease progression [11]. For this reason, we hypothesize that our data could reflect anti-oxidative mechanisms in the post-stroke brain for a long period after the ischemic event.

Our transcriptomic analysis showed upregulation of genes related to the retinoic acid pathway. *GPRC5A*, encoding for G Protein-coupled receptor (GPCR) class C group 5 member A (GPRC5A), also called RAIG1 (retinoic-acid-induced gene 1 protein) or RAI3 (Retinoic acid-induced protein). It is a member of the GPCR superfamily localized to the plasmatic membrane. *GPRC5A* is induced by all-trans-retinoic acid (atRA) [46]. There is evidence that *GPRC5A* is also induced in vivo during hypoxia, probably regulating the signaling that leads to the activation of hypoxia-adaptive genes [47]. In fact, its increase seems to be correlated with the expression of hypoxia-inducible factor (HIF) and the expression of *GPRC5A* protects cells from apoptosis in hypoxic conditions [48]. The s-arrestin gene, *SAG*, was found to be upregulated in our data. S-arrestin is mainly expressed and important in retinal photoreceptors and it is documented to play a crucial role in the regulation of GPCR signaling [49]. Even if there is no evidence of its specific interaction with GPRC5A, its strong upregulation could be related to this GPCR receptor.

*LRAT* codifies lecithin retinol acyltransferase (LRAT), an enzyme catalyzing the formation of retinyl esters by transferring an acyl group from the phosphatidylcholine (PC) into retinol (vitamin A) [50]. The neuroprotective effect of atRA has already been documented [51]. In fact, atRA is able to reduce neuroinflammation inhibiting the translocation of NF-κB and its consequent induction of inflammatory cytokine release [52]. Moreover, inflammation is reduced by retinoic acid through the induction of M2 anti-inflammatory macrophages and through reducing the production of tumor necrosis Factor-alpha (TNFα), interleukin 1β (IL-1β), and nitric oxide (NO) by microglia [53].

In addition, atRA is protective against ischemic stroke because it is able to recruit N2 anti-inflammatory neutrophils to the injured site after ischemic stroke [54]. Its neuroprotective role is also exerted through the modulation of the Bcl-2 protein and reduction of neuronal apoptosis [55]. For these reasons, the upregulation of the intermediate involved in the pathway of retinoic acids in samples of the non-acute stroke phase suggests that the neuroprotection exerted by atRA signaling and its related factors could still be present even years after the ischemic event.

Our results also showed upregulation of *ANXA1*, the gene encoding for annexin-A1. Protein Annexin-A1 binds phospholipids in a calcium-dependent manner. It interacts with the formyl peptide receptors (FPR), a G-coupled receptor, and through this, it exerts different functions related to proliferation, apoptosis, and differentiation [56]. Moreover, Annexin-A1 has immunomodulatory activity, recruiting neutrophils and monocytes [57]. It has already been demonstrated that Annexin-A1 has anti-inflammatory and protective action after ischemic stroke and it is also able to inhibit both acute and chronic inflammation, promoting M2 microglial polarization [58]. M2 microglial cells are the anti-inflammatory and neuroprotective phenotype [59], thus providing a neuroprotective effect after the ischemic injury [60].

Furthermore, the ischemic stroke immune response results in neutrophil and platelet recruitment, thus determining the production of pro-inflammatory and pro-thrombotic mediators and contributing to cerebrovascular injury [61]. It has already been documented that annexin-A1 is able to reduce platelet aggregation and their pro-thrombotic effect. Moreover, annexin-A1 promotes neutrophil recruitment and the phagocytosis of platelets [62], mitigating subsequent thromboinflammatory conditions [63].

Ansari et al. studied the protective role of annexin-A1 after ischemia–reperfusion injury. Annexin-A1 is able to reduce reperfusion-associated complications diminishing leucocyte adhesion and neutrophil recruitment and inhibiting the release of pro-inflammatory cytokines [64]. The upregulated expression of *ANXA1* emerging from our data suggests that this protein could have a protective role after ischemic stroke, reducing neuroinflammation and related brain damage even after an average of 2.6 years from the ischemic event. Furthermore, it seems that annexin-A1 could also mediate the anti-inflammatory action of retinoic acid; in fact, retinoic acid is able to enhance the expression of annexin-A1 and its receptor FPR2/ALX (N-formyl peptide receptor 2) [65], as mentioned before, in an important neuroprotective molecule.

The upregulation of *ANXA1* suggests that the anti-inflammatory, immunomodulatory, and anti-thrombotic activity of annexin-A1 could still be present, probably with the function to promote neuroprotection and mitigate brain damage, even when the patient is not in the acute phase anymore.

*BHLHE22*, downregulated in the stroke cohort, codifies for a protein belonging to the family of basic helix-loop-helix (bHLH) containing transcription factors. Bhlhe22 expression has been documented as almost limited to post-mitotic neurons and not in proliferating progenitors [66]. Its downregulation could probably be due to its role in a more mature stage and not in the initial phase of neural development or a mechanism related to neurological regeneration.

Non-coding genes *LINC00870*, *LINC01206*, *LINC01287*, and *LINC02073* are mainly more highly expressed in stroke patients, so they are upregulated. These are related to four long intergenic non-coding RNA (linc-RNA). LincRNAs are a class of long non-coding RNA (lncRNA), autonomously transcribed non-coding RNAs longer than 200 nucleotides and they are defined as “intergenic” because they lay between two protein-coding genes [67]. They have different functions such as: chromatin remodeling, RNA stabilization, being a scaffold for proteins, being molecular decoys, and transcriptome regulation [68]. It has already been documented that lincRNA are involved in ischemic stroke, in particular in the regulation of cell survival, inflammation, and angiogenesis. In fact, lncRNA are associated with the inhibition of inflammation and th esuppression of cell death [19]. Moreover, lncRNA are able to promote the autophagy of damaged and misfolded proteins in neurons, providing a neuroprotective effect [69].

In addition, their expression promotes angiogenesis which is fundamental to restoring circulation to the damaged area of the brain, improving the recovery of nervous function [70]. The upregulation of this lncRNA-related gene leads us to hypothesize that years after the ischemic events, lncRNA are still involved as neuroprotective factors, promoting neuronal regenerative processes in the brain. In addition, our results also showed upregulation of the genes *ENSG00000259363* and *ENSG00000273507*. These genes are related to long non-coding novel transcripts. Even if these are not yet well identified, their role could be comparable to the aforementioned linc-RNA.

Another gene that resulted in being upregulated is *SRPX2*. This codifies the Sushi repeat-containing protein X-linked 2 (SRPX2). The SRPX2 protein is expressed in the human brain, promoting synaptogenesis in the cerebral cortex [71]; it binds to urokinase the plasminogen activator receptor (uPAR) [72], regulating endothelial cells’ migration and angiogenesis [71,73].

Its high expression in our analyzed samples could be attributed to angiogenetic and pro-synaptogenesis processes in long-term neuronal recovery.

The observed strong upregulation of *F2RL2*, codifying for the protease-activated receptors 3 (PAR-3). PAR-3 is a high-affinity thrombin receptor, expressed in the cerebral cortex. Its role has been documented in post-ischemic injury, where it stimulate microglial cells to exert a neuroprotective role [74]. Its high expression suggest that this role is also present in the post-acute phase.

What is noteworthy is that for some DEGs, in our results, there are a few references about them but not their role in long-term recovery after ischemic stroke. We found upregulation of *DNAH2*, coding for dynein axonemal heavy chain 2. The dynein protein is the main component of the axoneme and its motility driving force [75]. Previous evidence has documented the probable expression of DNAH2 in the human brain [76], in which its functions are related to the active transport of signaling molecules along the axon [77]. We suggest that its long-term upregulation in an ischemic stroke affected brain is useful to contributing to the recovery of neuronal signaling.

The *MYO1G* gene codifies for the unconventional myosin-Ig, mainly described as a functional component of the phagocytic cup, and other cellular functions such as cell motility [78]. During an ischemic stroke, some of the microglia cells are activated and express the anti-inflammatory M2 phenotype of the phagocytes’ damaged and apoptotic cells [79]. *MYO1G* resulted in being upregulated in our analyzed samples and even if there is no evidence about its function in the neuronal environment, we suppose that Myo1G could be involved in phagocytosis occurring thanks to microglia recruitment after the ischemic event [80].

*EFHB* encodes for EF-hand domain-containing family member B and acts as a regulator of the store-operated Ca^2+^ entry (SOCE) [81]. This latter part is a fundamental mechanism for the control of cytoplasmic Ca^2+^ calcium signaling in the cell, also described as a regulator of neuronal activity [82]. Herein, *EFHB* resulted in being upregulated, so we can hypothesize that it could have a role in regulating correct neuronal activity during post-stroke recovery.

Our results also showed *PCDHA6* downregulation. This gene codifies for the protocadherin-α6 that seems to be involved in the creation of connections among brain cells with a pivot role. This protein has never been associated with stroke, but the cadherin family is located near to the membrane of synapsis and regulates the neural circuits [83]. We found *PCDHA6* downregulated, and we address this result to a diminution of creation of new synaptic interactions, a process that is probably present immediately after the event but not about two years later.

In addition, our in silico analysis showed up-regulation of *PKD1L2*, codifying for the polycystic kidney disease protein 1-like 2, and *PSORS1C1*, codifying for psoriasis susceptibility 1 candidate 1. These genes showed no correlation with nervous tissue and ischemic stroke.

**Figure 4:**
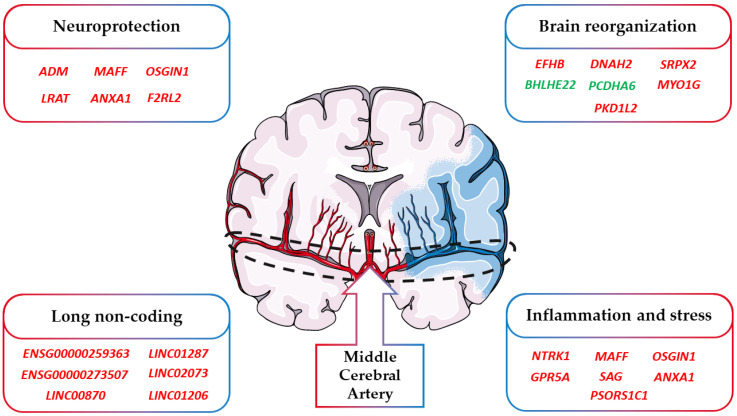
Classification of the DEGs involved in stroke. In the figure, we highlighted how the DEGs found in stroke can be included in the main categories “Neuroprotection”, “Brain reorganization”, “Inflammation and stress”, and “Long non coding”. Red DEGs are more expressed after stroke while green DEGs are less expressed after stroke.

A final consideration takes into account the cause of the ischemic stroke event of the patients. Indeed, the cause differs among the patients and their impact on our results. Specifically, the available information of our cohort points toward the attention of cardioembolic stroke. Indeed, for two of them, the stroke was caused by atrial fibrillation while for one of them, it was cryptogenic. Specifically, atrial fibrillation and cryptogenic strokes represent subtypes of cardioembolic stroke that are the consequence of emboli that occur in the heart and go through the brain from blood vessels [84]. As summarized in Figure 4, an ischemic stroke can modulate not only the genes involved in neuroinflammation and stress but also those involved in neuroprotection and brain reorganization. Nevertheless, strokes originating after other subtypes of cardioembolic or atherothrombotic strokes could have also activated other biological mechanisms.

In conclusion, our in silico analysis has highlighted that, after a period of about two years from the acute ischemic event, the genes involved in the neuroprotective role remain expressed.

## 5. Limitations

Along with the innovative findings of our study, we believe it noteworthy to point out some considerations. Indeed, as already mentioned, stroke is a multifactorial disease. For this reason, several pathological mechanisms can occur simultaneously, making the interpretation of the final outcomes more difficult. Although the comparative transcriptomic analysis was conducted, optimizing each step in order to make the analysis as reliable as possible based on the size of our cohort, having more samples may lead to interesting conclusions. This is also due to the variability of the causes of the stroke in our cohort. This consideration may lead us to consider the previous risk factors as peculiar to the individual along with their original ethnicity. We indeed cannot assume that the ethnicity is the same for all individuals of the cohort even if the brain samples are related to the same Swedish ethics committee. Additionally, the absence of neurological tests does not show as to whether the health status of the patients after the event is exactly the same. Finally, the lack of information for potential treatments taking place in the months or years after the stroke event and before the time of death may influence the level of the expression of the genes in the RNA-seq analysis.

## 6. Conclusions

The results obtained in this work highlight the presence of 23 DEGs in the brains of post-ischemic-stroke patients (who lived an average of 2.6 years) compared to healthy subjects. These coding and non-coding genes are involved in the modulation of anti-inflammatory and anti-oxidant processes.

We found this result interesting since neuroprotective genes appear to be expressed even years after the acute phase of ischemic stroke. Based on these findings, further studies should be conducted.

## Figures and Tables

**Table 1:** Cohort description.

Patient	Age		Gender		Death after Stroke	Cause of Stroke	Cause of Death of Stroke Patients
	Stroke	Non-Stroke	Stroke	Non-Stroke			
#1	76	Matched	F	Matched	5 years	Atrial fibrillation	Ischemic heart disease
#2	84	Matched	M	Matched	3 years	Not available	Congestive heart failure
#3	80	Matched	F	Matched	2 years	Cryptogenic	Myocardial infarction
#4	67	Matched	F	Matched	1 year	Not available	Sepsis
#5	75	Matched	M	Matched	2 years	Atrial fibrillation	Respiratory failure

The age was computed as the “year of death”–“year of birth”. In the gender column, F stands for female while M refers to male.

**Table 2:** DEGs identified in the comparative analysis between the healthy and stroke groups.

Gene	Healthy	Stroke	Fold Change	q-Value
*ADM*	20.77	349.11	4.11	4.19 × 10^−2^
*ANXA1*	45.43	221.86	2.2	4.46 × 10^−2^
*BHLHE22*	230.81	22.16	−3.41	2.10 × 10^−7^
*DNAH2*	40.6	172.2	2.14	4.46 × 10^−2^
*EFHB*	4.15	29.85	2.54	4.46 × 10^−2^
*ENSG00000259363*	3.69	32.32	2.8	4.19 × 10^−2^
*ENSG00000273507*	37.03	113.41	1.7	4.87 × 10^−2^
*F2RL2*	0.71	19.72	4.28	4.46 × 10^−2^
*GPRC5A*	1.04	29.22	4.36	4.87 × 10^−2^
*LINC00870*	2.24	24.69	3.12	4.87 × 10^−2^
*LINC01206*	1.02	35.84	4.69	4.87 × 10^−2^
*LINC01287*	0.25	22.85	5.54	4.87 × 10^−2^
*LINC02073*	6.43	38.1	2.28	4.87 × 10^−2^
*LRAT*	10.04	49.6	2.84	4.46 × 10^−2^
*MAFF*	20.89	168.54	3.06	4.87 × 10^−2^
*MYO1G*	4.34	53.96	3.32	4.46 × 10^−2^
*NTRK1*	0.85	27.31	4.45	4.46 × 10^−2^
*OSGIN1*	14.34	50.03	2.05	4.19 × 10^−2^
*PCDHA6*	95.99	32.11	−1.66	4.46 × 10^−2^
*PKD1L2*	24.64	114.37	2.21	4.46 × 10^−2^
*PSORS1C1*	2.79	27.34	2.97	4.46 × 10^−2^
*SAG*	1.13	25.75	4.03	4.87 × 10^−2^
*SRPX2*	5.2	53.61	3.07	4.87 × 10^−2^

All the values are rounded to the second decimal digit.

## Data Availability

The data presented in this study are openly available in the NCBI Sequence Read Archive at BioProject (accession number PRJNA242801).

## References

[1] Farina M., Vieira L.E., Buttari B., Profumo E., Saso L. (2021). The Nrf2 Pathway in Ischemic Stroke: A Review. Molecules.

[2] Feske S.K. (2021). Ischemic Stroke. Am. J. Med..

[3] Appelros P., Stegmayr B., Terént A. (2009). Sex differences in stroke epidemiology: A systematic review. Stroke.

[4] Hankey G.J. (2017). Stroke. Lancet.

[5] Jayaraj R.L., Azimullah S., Beiram R., Jalal F.Y., Rosenberg G.A. (2019). Neuroinflammation: Friend and foe for ischemic stroke. J. Neuroinflamm..

[6] Colonna M., Butovsky O. (2017). Microglia Function in the Central Nervous System During Health and Neurodegeneration. Annu. Rev. Immunol..

[7] Na K.S., Jung H.Y., Kim Y.K. (2014). The role of pro-inflammatory cytokines in the neuroinflammation and neurogenesis of schizophrenia. Prog. Neuro-Psychopharmacol. Biol. Psychiatry.

[8] Maida C.D., Norrito R.L., Daidone M., Tuttolomondo A., Pinto A. (2020). Neuroinflammatory Mechanisms in Ischemic Stroke: Focus on Cardioembolic Stroke, Background, and Therapeutic Approaches. Int. J. Mol. Sci..

[9] Zhu G., Wang X., Chen L., Lenahan C., Fu Z., Fang Y., Yu W. (2022). Crosstalk Between the Oxidative Stress and Glia Cells After Stroke: From Mechanism to Therapies. Front. Immunol..

[10] Haskew-Layton R.E., Payappilly J.B., Smirnova N.A., Ma T.C., Chan K.K., Murphy T.H., Guo H., Langley B., Sultana R., Butterfield D.A. (2010). Controlled enzymatic production of astrocytic hydrogen peroxide protects neurons from oxidative stress via an Nrf2-independent pathway. Proc. Natl. Acad. Sci. USA.

[11] Su X.T., Wang L., Ma S.M., Cao Y., Yang N.N., Lin L.L., Fisher M., Yang J.W., Liu C.Z. (2020). Mechanisms of Acupuncture in the Regulation of Oxidative Stress in Treating Ischemic Stroke. Oxidative Med. Cell. Longev..

[12] Kleinschnitz C., Grund H., Wingler K., Armitage M.E., Jones E., Mittal M., Barit D., Schwarz T., Geis C., Kraft P. (2010). Post-stroke inhibition of induced NADPH oxidase type 4 prevents oxidative stress and neurodegeneration. PLoS Biol..

[13] Wang L., Zhang X., Xiong X., Zhu H., Chen R., Zhang S., Chen G., Jian Z. (2022). Nrf2 Regulates Oxidative Stress and Its Role in Cerebral Ischemic Stroke. Antioxidants.

[14] Colucci-D’Amato L., Speranza L., Volpicelli F. (2020). Neurotrophic Factor BDNF, Physiological Functions and Therapeutic Potential in Depression, Neurodegeneration and Brain Cancer. Int. J. Mol. Sci..

[15] He Q., Ma Y., Liu J., Zhang D., Ren J., Zhao R., Chang J., Guo Z.N., Yang Y. (2021). Biological Functions and Regulatory Mechanisms of Hypoxia-Inducible Factor-1α in Ischemic Stroke. Front. Immunol..

[16] Eltzschig H.K., Eckle T. (2011). Ischemia and reperfusion—From mechanism to translation. Nat. Med..

[17] Zhang W., Zhu L., An C., Wang R., Yang L., Yu W., Li P., Gao Y. (2020). The blood brain barrier in cerebral ischemic injury—Disruption and repair. Brain Hemorrhages.

[18] Xie W., Zhou P., Sun Y., Meng X., Dai Z., Sun G., Sun X. (2018). Protective Effects and Target Network Analysis of Ginsenoside Rg1 in Cerebral Ischemia and Reperfusion Injury: A Comprehensive Overview of Experimental Studies. Cells.

[19] Bao M.H., Szeto V., Yang B.B., Zhu S.Z., Sun H.S., Feng Z.P. (2018). Long non-coding RNAs in ischemic stroke. Cell Death Dis..

[20] Qiu M., Zong J.B., He Q.W., Liu Y.X., Wan Y., Li M., Zhou Y.F., Wu J.H., Hu B. (2022). Cell Heterogeneity Uncovered by Single-Cell RNA Sequencing Offers Potential Therapeutic Targets for Ischemic Stroke. Aging Dis..

[21] Li Y., Zhang J. (2021). Animal models of stroke. Anim. Model. Exp. Med..

[22] Fluri F., Schuhmann M.K., Kleinschnitz C. (2015). Animal models of ischemic stroke and their application in clinical research. Drug Des. Dev. Ther..

[23] Barrett T., Wilhite S.E., Ledoux P., Evangelista C., Kim I.F., Tomashevsky M., Marshall K.A., Phillippy K.H., Sherman P.M., Holko M. (2013). NCBI GEO: Archive for functional genomics data sets—Update. Nucleic Acids Res..

[24] Huttner H.B., Bergmann O., Salehpour M., Rácz A., Tatarishvili J., Lindgren E., Csonka T., Csiba L., Hortobágyi T., Méhes G. (2014). The age and genomic integrity of neurons after cortical stroke in humans. Nat. Neurosci..

[25] Bolger A.M., Lohse M., Usadel B. (2014). Trimmomatic: A flexible trimmer for Illumina sequence data. Bioinformatics.

[26] Dobin A., Davis C.A., Schlesinger F., Drenkow J., Zaleski C., Jha S., Batut P., Chaisson M., Gingeras T.R. (2013). STAR: Ultrafast universal RNA-seq aligner. Bioinformatics.

[27] Anders S., Pyl P.T., Huber W. (2015). HTSeq—A Python framework to work with high-throughput sequencing data. Bioinformatics.

[28] Love M.I., Huber W., Anders S. (2014). Moderated estimation of fold change and dispersion for RNA-seq data with DESeq2. Genome Biol..

[29] Bagnato S. (2022). Biomarkers of Brain Injury: A Window on Mechanisms of Injury and Recovery in the Brain. Brain Sci..

[30] Ishiyama H., Tanaka T., Saito S., Koyama T., Kitamura A., Inoue M., Fukushima N., Morita Y., Koga M., Toyoda K. (2023). Plasma mid-regional pro-adrenomedullin: A biomarker of the ischemic penumbra in hyperacute stroke. Brain Pathol..

[31] Ferrero H., Larrayoz I.M., Gil-Bea F.J., Martínez A., Ramírez M.J. (2018). Adrenomedullin, a Novel Target for Neurodegenerative Diseases. Mol. Neurobiol..

[32] Lang M.G., Paternò R., Faraci F.M., Heistad D.D. (1997). Mechanisms of adrenomedullin-induced dilatation of cerebral arterioles. Stroke.

[33] Xia C.F., Yin H., Borlongan C.V., Chao J., Chao L. (2006). Postischemic infusion of adrenomedullin protects against ischemic stroke by inhibiting apoptosis and promoting angiogenesis. Exp. Neurol..

[34] Miyashita K., Itoh H., Arai H., Suganami T., Sawada N., Fukunaga Y., Sone M., Yamahara K., Yurugi-Kobayashi T., Park K. (2006). The neuroprotective and vasculo-neuro-regenerative roles of adrenomedullin in ischemic brain and its therapeutic potential. Endocrinology.

[35] Demir I.E., Tieftrunk E., Schorn S., Friess H., Ceyhan G.O. (2016). Nerve growth factor & TrkA as novel therapeutic targets in cancer. Biochim. Et Biophys. Acta.

[36] Fang C.N., Tan H.Q., Song A.B., Jiang N., Liu Q.R., Song T. (2022). NGF/TrkA promotes the vitality, migration and adhesion of bone marrow stromal cells in hypoxia by regulating the Nrf2 pathway. Metab. Brain Dis..

[37] Müller M.L., Peglau L., Moon L.D.F., Groß S., Schulze J., Ruhnau J., Vogelgesang A. (2022). Neurotrophin-3 attenuates human peripheral blood T cell and monocyte activation status and cytokine production post stroke. Exp. Neurol..

[38] Lu J., Wu D.M., Hu B., Zheng Y.L., Zhang Z.F., Wang Y.J. (2010). NGF-Dependent activation of TrkA pathway: A mechanism for the neuroprotective effect of troxerutin in D-galactose-treated mice. Brain Pathol..

[39] Garza-Lombó C., Petrosyan P., Tapia-Rodríguez M., Valdovinos-Flores C., Gonsebatt M.E. (2018). Systemic L-buthionine-S-R-sulfoximine administration modulates glutathione homeostasis via NGF/TrkA and mTOR signaling in the cerebellum. Neurochem. Int..

[40] Moon E.J., Mello S.S., Li C.G., Chi J.T., Thakkar K., Kirkland J.G., Lagory E.L., Lee I.J., Diep A.N., Miao Y. (2021). The HIF target MAFF promotes tumor invasion and metastasis through IL11 and STAT3 signaling. Nat. Commun..

[41] Wang X., Zhang Y., Wan X., Guo C., Cui J., Sun J., Li L. (2020). Responsive Expression of MafF to β-Amyloid-Induced Oxidative Stress. Dis. Mrk..

[42] Bellezza I., Giambanco I., Minelli A., Donato R. (2018). Nrf2-Keap1 signaling in oxidative and reductive stress. Biochim. Biophys. Acta Mol. Cell Res..

[43] Li R., Chen W., Yanes R., Lee S., Berliner J.A. (2007). OKL38 is an oxidative stress response gene stimulated by oxidized phospholipids. J. Lipid Res..

[44] Brennan M.S., Matos M.F., Richter K.E., Li B., Scannevin R.H. (2017). The NRF2 transcriptional target, OSGIN1, contributes to monomethyl fumarate-mediated cytoprotection in human astrocytes. Sci. Rep..

[45] Paul S., Candelario-Jalil E. (2021). Emerging neuroprotective strategies for the treatment of ischemic stroke: An overview of clinical and preclinical studies. Exp. Neurol..

[46] Hirano M., Zang L., Oka T., Ito Y., Shimada Y., Nishimura Y., Tanaka T. (2006). Novel reciprocal regulation of cAMP signaling and apoptosis by orphan G-protein-coupled receptor GPRC5A gene expression. Biochem. Biophys. Res. Commun..

[47] Bayat S., Mamivand A., Khoshnevisan A., Maghrouni A., Shabani S., Raouf M.T., Yaseri M., Saffar H., Tabrizi M. (2021). Differential Expression of Hypoxia-Related Genes in Primary Brain Tumors and Correlation with Clinicopathologic Data. World Neurosurg..

[48] Greenhough A., Bagley C., Heesom K.J., Gurevich D.B., Gay D., Bond M., Collard T.J., Paraskeva C., Martin P., Sansom O.J. (2018). Cancer cell adaptation to hypoxia involves a HIF-GPRC5A-YAP axis. EMBO Mol. Med..

[49] Mokarzel-Falcón L., Padrón-García J.A., Carrasco-Velar R., Berry C., Montero-Cabrera L.A. (2008). In silico study of the human rhodopsin and meta rhodopsin II/S-arrestin complexes: Impact of single point mutations related to retina degenerative diseases. Proteins.

[50] Golczak M., Kiser P.D., Sears A.E., Lodowski D.T., Blaner W.S., Palczewski K. (2012). Structural basis for the acyltransferase activity of lecithin:retinol acyltransferase-like proteins. J. Biol. Chem..

[51] Hummel R., Ulbrich S., Appel D., Li S., Hirnet T., Zander S., Bobkiewicz W., Gölz C., Schäfer M.K.E. (2020). Administration of all-trans retinoic acid after experimental traumatic brain injury is brain protective. Br. J. Pharmacol..

[52] Priyanka S.H., Syam Das S., Thushara A.J., Rauf A.A., Indira M. (2018). All Trans Retinoic Acid Attenuates Markers of Neuroinflammation in Rat Brain by Modulation of SIRT1 and NFκB. Neurochem. Res..

[53] Pouso M.R., Cairrao E. (2022). Effect of retinoic acid on the neurovascular unit: A review. Brain Res. Bull..

[54] Cai W., Wang J., Hu M., Chen X., Lu Z., Bellanti J.A., Zheng S.G. (2019). All trans-retinoic acid protects against acute ischemic stroke by modulating neutrophil functions through STAT1 signaling. J. Neuroinflamm..

[55] Kang J.B., Park D.J., Shah M.A., Koh P.O. (2021). Retinoic acid exerts neuroprotective effects against focal cerebral ischemia by preventing apoptotic cell death. Neurosci. Lett..

[56] Galvão I., de Carvalho R.V.H., Vago J.P., Silva A.L.N., Carvalho T.G., Antunes M.M., Ribeiro F.M., Menezes G.B., Zamboni D.S., Sousa L.P. (2020). The role of annexin A1 in the modulation of the NLRP3 inflammasome. Immunology.

[57] Kelly L., McGrath S., Rodgers L., McCall K., Tulunay Virlan A., Dempsey F., Crichton S., Goodyear C.S. (2022). Annexin-A1: The culprit or the solution?. Immunology.

[58] Zou J., Huang G.F., Xia Q., Li X., Shi J., Sun N. (2022). Electroacupuncture promotes microglial M2 polarization in ischemic stroke via annexin A1. Acupunct. Med. J. Br. Med. Acupunct. Soc..

[59] Guo S., Wang H., Yin Y. (2022). Microglia Polarization From M1 to M2 in Neurodegenerative Diseases. Front. Aging Neurosci..

[60] Xu X., Gao W., Li L., Hao J., Yang B., Wang T., Li L., Bai X., Li F., Ren H. (2021). Annexin A1 protects against cerebral ischemia-reperfusion injury by modulating microglia/macrophage polarization via FPR2/ALX-dependent AMPK-mTOR pathway. J. Neuroinflamm..

[61] De Meyer S.F., Denorme F., Langhauser F., Geuss E., Fluri F., Kleinschnitz C. (2016). Thromboinflammation in Stroke Brain Damage. Stroke.

[62] Senchenkova E.Y., Ansari J., Becker F., Vital S.A., Al-Yafeai Z., Sparkenbaugh E.M., Pawlinski R., Stokes K.Y., Carroll J.L., Dragoi A.M. (2019). Novel Role for the AnxA1-Fpr2/ALX Signaling Axis as a Key Regulator of Platelet Function to Promote Resolution of Inflammation. Circulation.

[63] Ansari J., Gavins F.N.E. (2021). Neutrophils and Platelets: Immune Soldiers Fighting Together in Stroke Pathophysiology. Biomedicines.

[64] Ansari J., Kaur G., Gavins F.N.E. (2018). Therapeutic Potential of Annexin A1 in Ischemia Reperfusion Injury. Int. J. Mol. Sci..

[65] Tsai W.H., Chien H.Y., Shih C.H., Lai S.L., Li I.T., Hsu S.C., Kou Y.R., Hsu H.C. (2012). Annexin A1 mediates the anti-inflammatory effects during the granulocytic differentiation process in all-trans retinoic acid-treated acute promyelocytic leukemic cells. J. Cell. Physiol..

[66] Ross S.E., McCord A.E., Jung C., Atan D., Mok S.I., Hemberg M., Kim T.K., Salogiannis J., Hu L., Cohen S. (2012). Bhlhb5 and Prdm8 form a repressor complex involved in neuronal circuit assembly. Neuron.

[67] Panzeri I., Rossetti G., Abrignani S., Pagani M. (2015). Long Intergenic Non-Coding RNAs: Novel Drivers of Human Lymphocyte Differentiation. Front. Immunol..

[68] Ransohoff J.D., Wei Y., Khavari P.A. (2018). The functions and unique features of long intergenic non-coding RNA. Nat. Rev. Mol. Cell Biol..

[69] Pan Y., Jiao Q., Wei W., Zheng T., Yang X., Xin W. (2021). Emerging Role of LncRNAs in Ischemic Stroke-Novel Insights into the Regulation of Inflammation. J. Inflamm. Res..

[70] Ren W., Yang X. (2018). Pathophysiology of Long Non-coding RNAs in Ischemic Stroke. Front. Mol. Neurosci..

[71] Miljkovic-Licina M., Hammel P., Garrido-Urbani S., Bradfield P.F., Szepetowski P., Imhof B.A. (2009). Sushi repeat protein X-linked 2, a novel mediator of angiogenesis. FASEB J..

[72] Tanaka K., Arao T., Tamura D., Aomatsu K., Furuta K., Matsumoto K., Kaneda H., Kudo K., Fujita Y., Kimura H. (2012). SRPX2 is a novel chondroitin sulfate proteoglycan that is overexpressed in gastrointestinal cancer. PLoS ONE.

[73] Liu K., Fan J., Wu J. (2017). Sushi repeat-containing protein X-linked 2 promotes angiogenesis through the urokinase-type plasminogen activator receptor dependent integrin αvβ3/focal adhesion kinase pathways. Drug Discov. Ther..

[74] Ye F., Garton H.J.L., Hua Y., Keep R.F., Xi G. (2021). The Role of Thrombin in Brain Injury After Hemorrhagic and Ischemic Stroke. Transl. Stroke Res..

[75] Maiti A.K., Mattéi M.G., Jorissen M., Volz A., Zeigler A., Bouvagnet P. (2000). Identification, tissue specific expression, and chromosomal localisation of several human dynein heavy chain genes. Eur. J. Hum. Genet. EJHG.

[76] Jones R.T., Abedalthagafi M.S., Brahmandam M., Greenfield E.A., Hoang M.P., Louis D.N., Hornick J.L., Santagata S. (2015). Cross-reactivity of the BRAF VE1 antibody with epitopes in axonemal dyneins leads to staining of cilia. Mod. Pathol..

[77] Cason S.E., Holzbaur E.L.F. (2022). Selective motor activation in organelle transport along axons. Nat. Rev. Mol. Cell Biol..

[78] Dart A.E., Tollis S., Bright M.D., Frankel G., Endres R.G. (2012). The motor protein myosin 1G functions in FcγR-mediated phagocytosis. J. Cell Sci..

[79] Jiang C.T., Wu W.F., Deng Y.H., Ge J.W. (2020). Modulators of microglia activation and polarization in ischemic stroke (Review). Mol. Med. Rep..

[80] Jia J., Yang L., Chen Y., Zheng L., Chen Y., Xu Y., Zhang M. (2021). The Role of Microglial Phagocytosis in Ischemic Stroke. Front. Immunol..

[81] Albarran L., Lopez J.J., Jardin I., Sanchez-Collado J., Berna-Erro A., Smani T., Camello P.J., Salido G.M., Rosado J.A. (2018). EFHB is a Novel Cytosolic Ca2+ Sensor That Modulates STIM1-SARAF Interaction. Cell. Physiol. Biochem. Int. J. Exp. Cell. Physiol. Biochem. Pharmacol..

[82] Courjaret R., Prakriya M., Machaca K. (2023). SOCE as a regulator of neuronal activity. J. Physiol..

[83] Hamada S., Yagi T. (2001). The cadherin-related neuronal receptor family: A novel diversified cadherin family at the synapse. Neurosci. Res..

[84] Akrawinthawong K., Venkatesh Prasad K., Mehdirad A.A., Ferreira S.W. (2015). Atrial Fibrillation Monitoring in Cryptogenic Stroke: The Gaps Between Evidence and Practice. Curr. Cardiol. Rep..

